# Endothelial *Epac1* facilitates YAP/TAZ controlled melanoma growth and angiogenesis

**DOI:** 10.1007/s10456-026-10080-6

**Published:** 2026-09-23

**Authors:** Yohanes Cakrapradipta Wibowo, Nan Ma, Yonggang Ren, Julio Cordero, Johannes Gahn, Zihao Chen, Magdalena Levay, Roxana Ola, Yuxi Feng, Gergana Dobreva, Harald Langer, Thomas Wieland, Christiane Vettel, Sepp Jansen

**Affiliations:** 1 Experimental Pharmacology Mannheim, European Center for Angioscience (ECAS), Mannheim Medical Faculty, Heidelberg University, Heidelberg, Germany; 2 Department of Cardiology, Angiology, Haemostaseology and Medical Intensive Care, European Center for Angioscience (ECAS), Medical Faculty Mannheim, University Medical Centre Mannheim, Heidelberg University, Mannheim, Germany; 3 Cardiovascular Systems Biology, Medical Centre Mannheim, Medical Faculty Mannheim, Heidelberg University, 68167 Mannheim, Germany; 4 Helmholtz-Institute for Translational AngioCardioScience (HI-TAC) of the Max Delbrück Center for Molecular Medicine in the Helmholtz Association (MDC) at Heidelberg University, 69117 Heidelberg, Germany; 5 German Centre for Cardiovascular Research (DZHK), Partner Site Heidelberg, Mannheim, Germany; 6 Center for Translational Medicine, the First Affiliated Hospital of Xi’an Jiaotong University, Xi’an, China; 7 Department of Cardiovascular Genomics and Epigenomics, European Center for Angioscience (ECAS), Medical Faculty Mannheim, Heidelberg University, Heidelberg, Germany; 8 Cardiovascular Pharmacology, European Center for Angioscience, Medical Faculty Mannheim, Heidelberg University, Heidelberg, Germany; 9 Institute of Experimental Cardiology, Internal Medicine VIII, Heidelberg University, Heidelberg, Germany

**Keywords:** Epac1, Tumor angiogenesis, Tumor endothelial cell, Melanoma, VEGFR2, YAP/TAZ, Mechanotransduction

## Abstract

The RapGEF Epac1 has emerged as an important modulator of pro-angiogenic signalling pathways, including VEGF/VEGFR2. However, its specific role in tumor angiogenesis has remained unexplored. Here, we identify endothelial Epac1 as a critical driver of melanoma angiogenesis. Analysis of patient-derived melanomas revealed that Epac1 is markedly upregulated in tumor endothelial cells. Using both global and endothelial-specific *Epac1* knockout mouse models, we demonstrate that *Epac1* deletion reduced melanoma growth and vascularization following subcutaneous melanoma cell inoculation. Transcriptome profiling of tumor-associated endothelial cells from *Epac1*-deficient mice showed broad downregulation of pro-angiogenic and YAP/TAZ target genes. This was corroborated in vitro using an *Epac1* knockout endothelial cell line, where VEGFR2 expression, YAP/TAZ nuclear translocation, and YAP/TAZ target gene transcription were all suppressed. Consistently, *Epac1* deletion impaired VE-cadherin/VEGFR2 interaction, VEGF-dependent nuclear YAP/TAZ and transcriptional activity, and abolished endothelial cell alignment in response to shear stress. Collectively, our results reveal Epac1 as a central modulator of VEGFR2 and YAP/TAZ signalling, integrating angiogenic and mechanosensory cues during melanoma vascularization.

**Supplementary Information:**

The online version contains supplementary material available at https://doi.org/10.1007/s10456-026-10080-6.

## Introduction

Formation of new vasculature from pre-existing blood vessels and remodelling of existing vasculature are tightly orchestrated processes essential during development and for tissue adaptation to changing oxygen and nutrient demands. The molecular mechanisms underlying angiogenesis and vascular remodelling are complex and context-dependent, but consistently require endothelial cell (EC) migration and proliferation, basement membrane formation, and recruitment of perivascular cells [1, 2]. Under pathological conditions, such as tumorigenesis, angiogenesis becomes dysregulated and contributes to tumor progression by supporting vascularization and growth [3]. In the tumor microenvironment, the vasculature is continuously remodelled, not only by pro- and anti-angiogenic factors secreted by tumor and stromal cells, but also by mechanical cues including extracellular matrix tension [1–4], interstitial pressure [5], and disturbed blood flow [3]. As a result, the tumor vasculature often exhibits an aberrant phenotype that further facilitates tumor progression and metastasis [3, 4]. Typically, the dysfunctional vasculature is characterized by high permeability, irregular and tortuous branching, and poor pericyte coverage, leading to fluid leakage and impaired perfusion [4, 6]. These features create hypoxic and acidic conditions, which further activate angiogenic signalling, most notably Vascular Endothelial Growth Factor (VEGF) [7, 8], and its receptor VEGFR2. VEGF/VEGFR2 signalling is a well-established driver of tumor angiogenesis [8–10]. In recent years, cAMP-mediated signalling has gained attention for its role in regulating tumor progression and angiogenesis [11–14]. Among the cAMP effectors, exchange protein directly activated by cAMP (Epac) has emerged as a promising therapeutic target in cancer [15, 16]. Epac exists in two isoforms, Epac1 and Epac2 [17], both acting as guanine nucleotide exchange factors (GEFs) for the small GTPase Rap1 (5). Notably, only Epac1 is expressed in EC [18, 19], where it participates in the regulation of various processes, including junctional dynamics and cell polarity [18, 20]. However, the pathophysiological function of endothelial Epac1 signaling is highly context-dependent. Consequently, both cell integrity and quiescence [21] as well as mitogenic activity and angiogenesis [16] are associated with increased Epac1 activity. We have previously shown that Epac1 promotes angiogenesis by regulating VEGFR2 expression [22]. Upon VEGF engagement, VEGFR2 undergoes autophosphorylation and activates multiple downstream pathways such as Ras/Raf/ERK, PI3K/Akt, and p38/MAPK [23]. Emerging evidence further suggests that VEGF/VEGFR2 signalling modulates transcriptional programs via the mechanosensitive Hippo pathway effector paralogues YAP and TAZ, which integrate extracellular signals and cytoskeletal dynamics to regulate endothelial function [24, 25]. These co-activators are activated in tumor endothelium and contribute to the pathological vascular remodelling [26].

Based on these findings and our previous work linking Epac1 to VEGF/VEGFR2 signalling, we hypothesized that endothelial Epac1 plays a key role in tumor vascularization. Here, we demonstrate that Epac1 is essential for melanoma vascularization and provide the first evidence that Epac1 regulates YAP/TAZ-mediated transcription in the tumor vasculature.

## Materials and methods

### Animal experiments

Animal experiments were performed in accordance with the European Community guiding principles of care and use of animals (2010/63/EU). Authorization for animal experiments was obtained from Regierungspräsidium Karlsruhe, Germany (animal protocols G-178/15 and G-164/20). The mice used in this study had a C57BL/6N background and were bred and maintained in the animal core unit of the Medical Faculty Mannheim of Heidelberg University. We used male adult mice at the age of 12 weeks for the global *Epac1*^KO/KO^ study, and female mice for the endothelial-selective *Epac1* knockout mice study. *Epac1*^KO/KO^ global knockout mice were generated as previously described [27]. Inducible endothelial-selective *Epac1* knockout mice were generated by crossing *Epac1*^flox/flox^ and *Cdh5*-*Cre*^ERT2^. Both the *Epac1*^KO/KO^ and *Epac1*^flox/flox^ mice were a kind gift from Prof. Frank Lezoualc’h, I2MC Institute, whereas the *Cdh5-Cre*^ERT2^ mice were a kind gift from Prof. Ralf Adams, Max Planck Institute. To induce *Epac1* endothelial-selective knockout, intraperitoneal tamoxifen (75 mg/kg BW/day) (Sigma Aldrich, T5648) injection was performed for 5 consecutive days. Tamoxifen was prepared by dissolving in corn oil (Sigma Aldrich, C8267) and shaking at 37 °C overnight covered in aluminum foil.

## Subcutaneous tumor inoculation

Animals received a single subcutaneous injection of 0.5 × 10^6^ B16F10 melanoma cells in 100 µl phosphate-buffered saline (PBS) on the lateral flank. Female animals were housed in groups of 4, whereas male animals were housed individually to avoid fighting. Tumor growth was assessed by measuring the tumor with a caliper on days 10, 12, 14, 16, and 18 (only in global *Epac1* KO mice) after tumor cell inoculation. The volume was calculated using the formula V = 0.5L x W^2^, in which V = volume, L = length, W = width. The study was terminated based on predefined humane endpoints. In accordance with animal regulations, no pre-mature mortality occurred during tumor growth.

## Mice brain endothelial cell isolation

Mice brain EC (MBEC) isolation was performed by magnetic-activated cell sorting (MACS) according to manufacturer’s recommendations. Brain were dissected immediately after sacrifice by making a careful incision in the cranium from orbital area to foramen magnum. The brain was removed by carefully flipping the cranial bone laterally without damaging the brain and lifting the brain out with a round spatula. Cerebellum and olfactory bulbs were removed. The brain was placed on a sterile filter paper and rolled to remove meninges. Brains were collected into C-tube containing Collagenase/Dispase (Sigma Aldrich, 10269638001) and DNAse I (Sigma Aldrich, DN25) in Hank’s balanced salt solution (HBSS) without Ca^2+^ and Mg^2+^, and processed in a MACS dissociator (Miltenyi Biotec, 130–093-235), put through a 70 µm cell strainer (Corning, 431751), and washed with PEB buffer (0.5% BSA, 2 mM EDTA in PBS). Myelin was removed by centrifugation at 2000 × g for 30 min in 20% BSA in HBSS at 4 °C. Leukocytes were negatively selected by incubating with CD45 beads (Miltenyi Biotec, 130–052-301) on LD column (Miltenyi Biotec, 130–042-901) in the QuadroMACS separator (Miltenyi Biotec, 130–090-976). Flow through was subsequently incubated with CD31 beads for EC enrichment (Miltenyi Biotec, 130–097-418) on MS column (Miltenyi Biotec, 130–042-201) and separated by OctoMACS separator (Miltenyi Biotec, 130–042-109). Cells were further selected for 2 days with 4 μg/ml puromycin (Sigma-Aldrich, P8833).

## Tumor endothelial cell isolation

Tumor EC (TEC) isolation was performed as previously described [28]. 16 days after tumor inoculation, mice were sacrificed, and tumors were excised. 3 tumors were pooled and diced with a scalpel and homogenized in HBSS with Ca^2+^ and Mg^2+^ containing 10 mg/mL collagenase Type II (Worthington, LS004176) and 25 μg/ml DNase I (Sigma-Aldrich, DN25). Digestion was performed for 30 min at 37 °C in a rotating vessel. Cells were layered on Histopaque (Sigma-Aldrich, 10771) and centrifuged at 300 × g for 20 min at 22 °C. The buffy coat was transferred into a new 50 ml tube containing HBSS without Ca^2+^ and Mg^2+^ and cells were centrifuged at 400 × g for 10 min at 4 °C. Leukocytes negative selection was performed by using CD45 MicroBeads (Miltenyi Biotec, 130–052-301) on LD column (Miltenyi Biotec, 130–042-901) in the QuadroMACS separator (Miltenyi Biotec, 130–090-976). TEC population enrichment was done by using CD31 MicroBeads (Miltenyi Biotec, 130–097-418) for positive selection on MS column (Miltenyi Biotec, 130–042-201) and separated by OctoMACS separator (Miltenyi Biotec, 130–042-109). Subsequently, cells were stained with CD31 (BD Biosciences, 551262) antibody for FACS sorting. Dead cells were filtered out using SYTOX™ Red Dead Cell Stain (Invitrogen, S34859). TEC were defined as CD31 + ; CD45-.

## HUVEC isolation

Human Umbilical Vein EC (HUVECs) were isolated from fresh human umbilical cords obtained from University Hospital Mannheim (UMM) under authorization (2012-388N-MA) from *Ethik-Kommission II der Universität Heidelberg* as previously described [29]. Briefly, HUVECs were isolated by digestion with 1 mg/ml dispase into the lumen of the umbilical vein and incubated for 30 min at 37 °C and 5% CO2. Cells were suspended in DMEM containing 10% foetal bovine serum (FBS) (Sigma-Aldrich, F7524) to quench dispase. HUVECs were seeded in EC growth medium (PromoCell, C-22110) containing EC growth supplements (PromoCell, C-39210) and 1% penicillin/streptomycin (Sigma-Aldrich, P4333).

## Cell culture and treatment

Primary HUVEC and bEnd.3 cells were cultured in EC growth medium containing supplements, 1% penicillin/streptomycin with 2% and 10% (v/v) FBS, respectively. Unless otherwise specified, for every experimental purpose, bEnd.3 cells were serum-deprived for 48 h in 0.5% FBS prior to any treatment. HUVEC and bEnd.3 were cultured on 0.25% gelatin-coated dishes and were used until passage 4 and 35, respectively. B16F10 melanoma cells were cultured in RPMI 1640 Medium (Gibco, 11875093) containing 10% (v/v) FBS (Sigma, F7524), 1% penicillin/streptomycin and 1% L-glutamine (Sigma, G7513). MLE12 cells were cultured in DMEM (Sigma-Aldrich, D-6546) supplemented by 10% (v/v) FBS (Sigma, F7524), 1% penicillin/streptomycin and 1% L-glutamine. All cells were maintained at 37 °C and 5% CO_2_. Recombinant human VEGF (#BT-VEGF-050) was purchased from Bio-Techne. CT04 (#CT04-A) was obtained from Cytoskeleton, Inc. Anlotinib (#S8726) was from Selleckchem. CE3F4 (#4793) was purchased from Tocris.

## RNA sequencing and analysis

TEC were lysed with RLT Buffer (Qiagen), and total RNA was isolated according to the manufacturer’s recommendations without carrier RNA (RNeasy Micro Kit, 74004, Qiagen). The low input RNA samples were sent for RNA sequencing based on a Switching Mechanism at the 5′ end of RNA Template (SMART) method. RNA from bEnd.3 cells was isolated using Trizol followed by RNA purification steps according to manufacturer’s recommendations (DirectZol, R2052, Zymo). RNA samples were processed for RNA sequencing by using combinatorial probe-anchor synthesis (cPAS) method. RNA-seq reads were mapped to mice GRCm39 by using Spliced Transcripts Alignment to a Reference (STAR) [30]. Differential expressions were measured and normalized by using DESeq2. Gene ontology (GO) analysis was performed by using goseq [31] in Galaxy [32] and was limited to GO “biological process”. For TEC transcriptome, genes with an FDR adjusted p value (q value) < 0.05 and a log2fold change of > 0 or <  − 0 were considered significantly upregulated and downregulated, respectively. To identify shared differentially expressed genes between TEC and bEnd.3, we applied an FDR-adjusted* p*-value < 0.05 in both datasets, using a log₂fold change of > 0 or < 0 for TEC, and > 0.58 or <  − 0.58 for bEnd.3.

## Analysis of single cell RNA seq data from primary human melanoma tumors

We used single-cell RNA-seq data that was deposited online after initially being published [33, 34]. Raw data from primary human melanoma were obtained from GSE277165, and data from healthy human skin (used as a control) were downloaded from GSE243940. All datasets were processed using the Seurat R package (version 5.1.0) [35]. Quality control filtering was performed to exclude low-quality cells with low feature counts or high mitochondrial content. The data were normalized using SCTransform and principal component analysis (PCA) was applied for dimensionality reduction. Graph-based clustering was done and visualized using uniform manifold approximation and projection (UMAP). Cell types were annotated based on canonical marker expression. Endothelial cell clusters were identified from each dataset by co-expression of *CDH5* (VE-cadherin), *PECAM1* (CD31), and *VWF*. After extraction, batch effects were removed, and melanoma and control datasets were integrated following Seurat guidelines. Statistical analyses were performed using the ggpubr R package (version 0.6.0) [36].

## Expression vectors

*Epac1* was deleted using CRISPR/Cas9. sgRNAs were designed by using chop-chop (https://chopchop.cbu.uib.no/) [37]. sgRNAs were cloned into the TLCV2 vector and were validated in MLE12 cells by using a mutation detection kit (Takara Bio, 631443). The two most efficient sgRNAs (sgRNA *Epac1* L1: ACCAGCTAGTGTTCGAGCAC and sgRNA *Epac1* L4: TCGGATGAGGGTAGGGTACG) were selected for further experiments. For *Epac1* overexpression, *Epac1* cDNA was cloned into the ADR3 IRES plasmid backbone. This plasmid was generated based on stem cell cassette (StemCCA). StemCCA construct was replaced by multiple cloning site (MCS) which also called ADR3, internal ribosomal entry site (IRES), a puromycin resistance gene (PuroR) and a red fluorescent protein (RFP) sequence [38].

## Lentiviral preparations

Indicated constructs were packed into 3rd generation lentiviral packaging system (pMD2.G, pMDLg/pRRE, pRSV-Rev [39]) and produced in HEK293T cells (CRL-3216, ATCC). Transfection was performed using Lipofectamine 3000 (Thermosfisher, L3000015) and Lipofectamine LTX (Thermosfisher, 15338100) as described previously [40]. 10 mM sodium butyrate was added to increase viral titer. Supernatant was collected at 24 h, 48 h, and 72 h, and viral particles were collected using Vivaspin® 6 columns (Sartorius, VS2041). Concentrated virus was stored in single-use aliquots at − 80 °C until further use. For viral transduction, 5 × 10^4^ bEnd.3 were seeded per well in a 12-well plate. Transduction was done for 48 h in the presence of 4 µg/ml polybrene (Sigma-Aldrich, TR-1003) at a MOI of 100. Subsequently, transduced cells were selected with 2 µg/ml puromycin for 48 h. Both *CRISPR ctrl* and *Epac1 KO* cell lines originated from pooled cell populations.

## qRT-PCR

A total of 1 µg RNA was used for first-strand cDNA synthesis using GoScript™ Reverse Transcriptase—cDNA Synthesis (Promega, A5003). qRT-PCR was performed by using a reaction master mix (Kapa Biosystems, KK4705) and the TaqMan probes for mice *Epac1* (Thermofisher, Mm01303434_g1), mice *Ctgf* (Thermofisher, Mm01192933_g1), mice *Vegfr2* (Thermofisher, Mm01222421_m1), mice *Ppia* (Thermofisher, Mm02342430_g1) human *CTGF* (Thermosfisher, Hs00170014_m1), human *CYR61* (Thermosfisher, Hs00155479_m1), human *ANKRD1* (Thermosfisher, Hs00173317_m1), and human *HPRT* (Thermosfisher, Hs02800695_m1). Thermocycling was performed on Quantstudio 3 (Thermosfisher, A28567). Relative changes in gene expression were calculated according to the ΔΔCt method.

## Plasmid transfection

Cells were transfected with plasmid DNA at 70% confluency in a serum- and antibiotics-free EC growth medium. Cells were transfected with 50% plasmid DNA of pEGFP-C3-hYAP1 (Addgene, 17843) [41] and 50% of pLL3.7-EGFPC2-TAZ (Addgene, 66850) [42] by using Lipofectamine 3000 (Thermofisher, L3000015) according to manufacturer’s protocol. For luciferase reporter gene assays, cells were transfected with luciferase reporter constructs 2.5 µg (in a 6 well plate format) mixed of HOPFlash, HIPFlash [43] and renilla pRL-TK by using Lipofectamine 3000 in 65% confluency according to manufacturer’s recommendations. After 72 h, firefly and renilla luciferase activity was measured in a plate reader (Perkin Elmer, Envision 2102) by using the Dual Reporter Luciferase Assay System (Promega; E1960) according to the manufacturer’s recommendations.

### SDS-PAGE

Cells were lysed in RIPA buffer (50 mM Tris, pH 7.4, 1% Triton X-100, 150 mM NaCl, 1 mM EDTA pH 8.0, 1% sodium deoxycholate containing cOmplete™ Protease Inhibitor Cocktail (Roche, 11697498001) and PhosSTOP™ phosphatase inhibitors (Roche, 4906837001). Proteins were then denatured with Laemmli buffer (62.5 mM Tris–HCl, 20% glycerol, 2% SDS, 5% β-mercaptoethanol, pH 6.8) and boiled at 95 °C for 5 min. Proteins were separated in acrylamide gels and transferred to nitrocellulose membranes (Amersham, GE10600000). Membranes were blocked with Roti® Block (Carl Roth) for 1 h at room temperature. Membranes were incubated with primary antibodies overnight at 4 °C in TBS (10 mM Tris, 150 mM NaCl pH: 7.4) containing 1% Tween20 (TBST) followed by incubation with species-appropriate HRP-conjugated secondary antibodies. The immuno-reactive bands were visualized with chemiluminescence (ThermosFisher, 34,096) and then quantified by densitometry analysis using ImageJ software. The following primary antibodies were used for western blots: CTGF (1:500; Santa Cruz, sc-365970), Epac1 (5D3) (1:500; Cell Signaling, 4155), GAPDH (1:1000; Santa Cruz, sc-47724), MLC2 (1:1000; Cell signaling, 3672), phospho-MLC2 (1:1000; Cell signaling, 3675), phospho-VE-cadherin Y658 (1:1000; Invitrogen, 44–11-44G), RhoA (1:200; Santa Cruz, sc-418), TAZ (1:500; BD Biosciences, 560235), Tubulin (1:1000; Sigma-Aldrich, T6557), VE-cadherin (1:1000; Abcam, ab205336), VEGFR2 (1:1000; Cell Signaling, 2479), YAP (1:1000; ThermoFisher, PA1-46189), HRP-conjugated anti-mouse (1:5000; Sigma-Aldrich, A9044), and HRP-conjugated anti-rabbit (1:5000; Sigma-Aldrich, A9169).

## Immunofluorescence

Cells were grown on µ-Slide 15-well slides (IBIDI, 81,506), fixed with 4% PFA, and permeabilized with 0.1% v/v Triton X-100. After blocking with 1% w/v BSA and 2% v/v goat serum, primary antibodies were incubated overnight at 4 °C. The following primary antibodies were used: YAP (1:500; ThermoFisher, PA1-46189), TAZ (1:100; BD Biosciences, 560235), VE-cadherin (1:1000; Abcam, ab205336), Phalloidin-TRITC (1:1000; Sigma-Aldrich, P1951) or Ki67 (1:250; Abcam, ab16667-500) for proliferation assay. Next day, cells were incubated with fluorophore-conjugated species-appropriate secondary antibodies at room temperature for 60 min. Species-appropriate Alexa Fluor 488 or Alexa 594 conjugated secondary antibodies (Life Technologies, A-11008, A-11032) were used. Nucleus was visualized using counterstaining with 4′,6-diamidino-2-phenylindole (DAPI) (1:1000; Sigma-Aldrich, D9542).

Tumors were embedded in OCT (Sakura, 4583) and were frozen at -80 °C. Tissue sections of 7 µm were cut and stored at -80 °C until further use. Before use, sections were fixed with acetone (-20 °C) and washed twice for 10 min with 1% goat serum in PBS with 0.4% Triton X-100 (PBST), followed by blocking for 30 min with 5% goat serum in PBST at room temperature. Antibodies were diluted in PBST containing 1% goat serum and incubated overnight at 4 °C. Secondary antibody incubation was performed for 1 h at room temperature. After washing, slides were stored in ROTIMount Fluorecare anti-fade mounting medium (Carl-Roth, HP19.1). The following primary antibodies were used: CD31 (1:400; BD Biosciences, 551,262), YAP/TAZ (1:200; Cell Signaling, 8418), VEGFR2 (1:100; Cell Signaling, 2479).

## Fluorescence in situ hybridization

Single molecule Fluorescence In Situ Hybridization (smFISH) was performed according to manufacturer’s recommendations (Pixelbiotech). Briefly, a thin layer (7 µm thickness) from cryo-preserved tissue was fixed with 3% PFA/PBS at room temperature for 10 min. Slides were incubated overnight with 70% Ethanol at 4 °C, after which they were washed twice with 2xSSC, 2 M Urea (HuluWash) for 2 × 10 min. Hybridization with HuluFISH probe was performed by incubating the slides overnight in a humidified chamber at 30 °C, followed by washing with HuluWash for 10 min at room temperature for a total of 4 times. The slides were covered with ROTIMount Fluorecare anti-fade mounting medium (Carl-Roth, HP19.1). Nucleus was counterestained with 4′,6-diamidino-2-phenylindole (DAPI) (1:1000; Sigma-Aldrich, D9542).

## Proximity ligation assay

Cells were grown on µ-Slide 15-well slides (IBIDI, 81506) or µ-Slide VI 0.4 (IBIDI, 80606) for shear stress exposure (described below). Cells were fixed with 4% PFA and proximity ligation was performed by NaveniFlex GR kit (Navinci, NC.GR.100) according to manufacturer`s recommendations, using VE-cadherin (1:1000; Abcam, ab205336) and VEGFR2 (1:100; R&D Systems, AF644) antibodies to detect the interaction between the two proteins. Nucleus was counterstained with 4′,6-diamidino-2-phenylindole (DAPI) (1:1000; Sigma-Aldrich, D9542).

## Angiogenic sprouting assay

In vitro sprouting angiogenesis assays with bEnd.3 were performed as described previously [44]. Briefly, 400 cells in medium containing methylcellulose were pipetted as hanging drops onto a culture dish (Greiner Bio One International, 688102), which was then incubated in a humidified 5% CO_2_ incubator for 24 h at 37 °C to generate spheroids. The spheroids were then embedded into a collagen gel matrix mixed with methyl cellulose containing 10% FBS without supplements and followed by treatment with the indicated compounds for 24 h.

## Monomeric GTPase activity assay

Guanosine triphosphate (GTP)-bound RhoA was quantified using pulldown assays as described previously [45, 46]. This assay measured RhoA-GTP bound to GST fusion protein containing the Rho-binding domain of rhotekin (GST-RBD). Rhotekin was expressed and purified in *E. coli*. Briefly, bEnd.3 were lysed in ice-cold GST-FISH buffer (50 mM Tris–HCl, 10% glycerol, 100 mM NaCl, 1% NP-40, 2 mM MgCl_2_, pH 7.4). GTP-bound monomeric GTPases were pulled down with the Rho binding domain (RBD) of rhotekin coupled to glutathione sepharose. The amounts of active (GTP-bound) and total RhoA were then determined by SDS-PAGE.

## Flow-induced shear stress

Cells were grown on µ-Slide VI 0.4 (IBIDI 80606) in the presence of 5% FBS and 1% penicillin/streptomycin without supplements. µ-Slides were installed into an IBIDI Pump System (IBIDI, 10906) set up for oscillatory shear stress (12 dyne/cm2 at 2 Hz). After 24 h, cells were fixed with 4% PFA and further processed for immunofluorescence staining.

## Permeability assay

Endothelial permeability assay was performed by monitoring the passage of FITC-labeled 70-kDa dextran through a monolayer of HUVECs. HUVECs were seeded on Transwell cell culture inserts with a pore size of 3 μm (Corning, 3462) until fully confluent and then 0.5% serum starved overnight and pre-incubated with CE3F4. After CE3F4 pre-incubation, 1 mg/ml FITC-dextran (Sigma-Aldrich, FD70S) was added to the upper Transwell compartment. A sample from the bottom compartment was taken to measure passage of FITC-dextran through the monolayer using a multi-label plate reader (Perkin Elmer, Envision 2102) by measuring the fluorescence intensity (Ex/Em: 485/535 nm).

## Microscopy and image analysis

Images were taken using Zeiss LSM700 confocal microscope with Plan-Apochromat 10 × /0.45 objective, Plan-Apochromat 20 × /0.8 NA Ph2 objective and W Plan-Apochromat 63 × /1.0 objective. Images were taken using Zen 2.3 software (Zeiss) with z-stacks consisting of 5–10 optical slices at 1 µm at room temperature. All image analysis was done by ImageJ. Images were taken at minimum five randomly selected regions per replicate.

## Image quantification

Quantification of vessel density was performed using ImageJ by quantifying the CD31-positive area as a percentage of the total area. Quantification of VEGFR2 in the tumor vasculature was performed in ImageJ by quantifying the VEGFR2 signal intensity in the CD31-positive area. Quantification of *Ctgf* in tumor sections was performed by counting the *Ctgf* smFISH dots within the CD31 positive area. For each tumor image quantification, 5 randomly selected fields per tumor were analysed. The quantification of YAP/TAZ localization in bEnd.3 cells was performed by quantifying the relative signal intensity of YAP/TAZ in the DAPI-positive (nuclear) area and the DAPI-negative (cytosolic) area. For quantification of VE-cadherin/VEGFR2 co-localization, PLA-positive dots were quantified using the Spots detection and Colocalization (ComDet) plugin (https://imagej.net/plugins/spots-colocalization-comdet) in ImageJ. Quantification of F-actin was performed by analysing the number of actin stress fibers using ImageJ. The number of actin filaments was quantified by the number of peaks from an intensity plot derived from a cross-section. For analysis of cell alignment, OriginPro software (OriginLab, MA, USA) was used to generate windrose diagrams. Orientation of bEnd.3 cells was measured by the angle of the vector of the flow and longitudinal axis of the cell was quantified. Aligned was defined as an absolute value of angle between 0 and 45°, not aligned as an absolute value of angle between 45 and 90°. For cell stainings, a minimum of 30 cells in 5 different fields were analyzed. Sprouting was quantified by measuring the length of each sprout of at least 10 spheroids using ImageJ. Cumulative sprout length is the cumulative length of all sprouts originating from one spheroid.

Ki67 immunofluorescence staining was used to assess endothelial cell proliferation. For each condition, Ki67-positive nuclei and total DAPI-stained nuclei were quantified from randomly selected microscopic fields using ImageJ. The Ki67 proliferation index was defined as the percentage of Ki67-positive nuclei among total nuclei and calculated as: Ki67 proliferation index (%) = Ki67-positive nuclei / total DAPI-positive nuclei × 100.

## Statistical analysis

All results are presented as mean ± SEM. Data from two groups were analysed by using unpaired, one-tailed Student’s t-test. Comparisons of three and more groups were performed with ANOVA and Tukey’s HSD post-hoc test. Mann-Whitney and Kruskal–Wallis were used to analyse non-parametric data. All statistical analysis was performed in GraphPad Prism 10.

## Results

### Global loss of *Epac1* leads to a reduction in subcutaneous melanoma tumor volume and to impaired melanoma angiogenesis

**Fig. 1:**
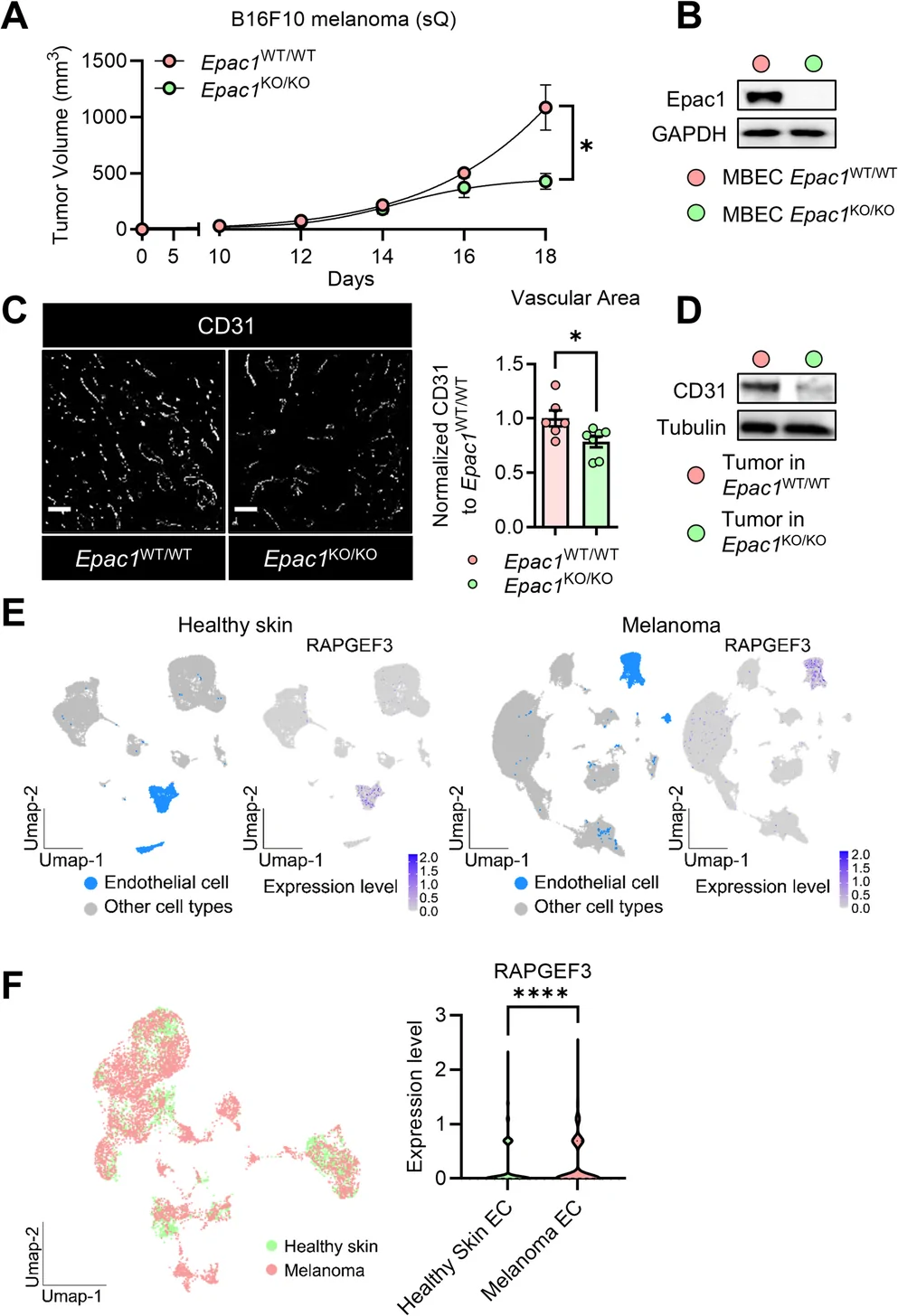
Global deletion of Epac1 decreases tumor growth and angiogenesis ** A**. B16F10 melanoma tumor volume in *Epac1*^WT/WT^ and *Epac1*^KO/KO^ mice. The x-axis represents the days since tumor cell inoculation, N = 6–7 per group. **B**. Representative western blot images of Epac1 expression in MBEC isolated from *Epac1*^WT/WT^ and *Epac1*^KO/KO^ mice and B16F10 melanoma cells used for tumor cell inoculation. **C**. Representative images of tumor vascularization of B16F10 subcutaneous melanoma tumors excised from *Epac1*^WT/WT^ and *Epac1*^KO/KO^ mice. Vascularization was visualized with CD31 staining. The total vascular area was measured. Data represent the average CD31-positive area for each tumor and are the mean ± SEM of 6–7 mice per group and are normalized to the average vascular area from *Epac1*^WT/WT^. Scale bars, 20 µm. **D**. Representative western blot images of expression of CD31 in melanoma tissue excised from *Epac1*^WT/WT^ and *Epac1*^KO/KO^ mice. **E**. Uniform Manifold Approximation and Projection (UMAP) of all cells in healthy human skin and primary human melanoma tissue analysed by scRNA-seq. Blue cells indicate cells expressing EC markers. Purple represents *RAPGEF3* positive cells. **F**. UMAP visualization of integrated EC population from healthy skin (green) and human melanoma (red) demonstrating the absence of a batch effect on EC gene expression in both datasets. Comparison of *RAPGEF3* expression in melanoma EC vs healthy skin EC. * p < 0.05, ** p < 0.01, **** p< 0.0001.

In recent years, the contribution of Epac1 to tumor growth and progression has become increasingly evident from studies targeting Epac1 in malignant cells [15, 47–49]. However, considering the importance of Epac1 in the endothelium, its role in stromal cells that constitute the tumor microenvironment remains unclear. To investigate the impact of Epac1 on tumor growth [27], mice with a global *Epac1* deletion (*Epac1*^KO/KO^) were subjected to a subcutaneous melanoma model by inoculating B16F10 cells in the flank. Tumor volume was measured every second day after inoculation until day 18, when several animals reached predefined humane endpoint. Palpable tumors developed in both *Epac1*^WT/WT^ and *Epac1*^KO/KO^ animals by day 10; however, melanoma growth in *Epac1*^KO/KO^ animals was markedly reduced after day 14, resulting in an approximately 50% decrease in tumor size by day 18 compared with *Epac1*^WT/WT^ mice (Fig. 1A). Endothelial Epac1 deletion was confirmed in isolated brain endothelial cells (MBEC) from *Epac1*^KO/KO^ mice as a representative vascular bed, consistent with global Epac1 deletion across tissues (Fig. 1B). Of note, the plateau in melanoma growth in *Epac1*^KO/KO^ mice coincided with the stage at which vascularization becomes growth limiting, suggesting that melanoma vascularization might be impaired. To assess this, tumor sections were stained for the endothelial marker CD31. The CD31-positive area was significantly smaller in melanoma from *Epac1*^KO/KO^ mice (Fig. 1C), and CD31 expression was likewise reduced in tumor lysates (Fig. 1D). These findings demonstrate that global loss of *Epac1* leads to impaired melanoma vascularization.

## Epac1 expression is increased in human melanoma endothelial cells

To examine Epac1 expression in human tissue, we analysed publicly available single-cell RNA sequencing (scRNA-seq) datasets from human healthy skin (GSE243940 [33]) and human primary melanoma (GSE277165 [34]). EC clusters were identified based on the expression of canonical EC markers *CDH5*, *PECAM1*, and *VWF* (Fig. S1A). Two distinct EC populations originating from vascular (large cluster) and lymphatic (*LYVE1*, *PROX1*, *FLT4* positive; small cluster, Fig. S1B) vessels were clearly resolved in UMAP projections (Fig. 1E, blue cells). A full annotation of all identified cell clusters and respective marker genes are given in Fig. S2A-C, confirming the presence of all major cell types within the skin and melanoma tissue samples (Fig. S2B-C). *RAPGEF3* (*EPAC1*) was predominantly expressed in vascular EC compared to lymphatic and compared to non-endothelial cells from both healthy skin and primary melanoma tumors (Fig. 1E, purple dots, Fig. S1C, *RAPGEF3* plot) suggesting that the attenuated melanoma growth and vascularization in *Epac1* KO animals was likely the result of endothelial *Epac1* deficiency and not from other stromal cells. Integration of both datasets confirmed the absence of batch effects (Fig. 1F, UMAP plot). The combined analysis revealed that *RAPGEF3* expression was markedly enriched in melanoma-derived EC compared to EC from healthy skin (Fig. 1F, *RAPGEF3* plot), indicating upregulation of Epac1 in melanoma endothelium.

## Endothelial Epac1 is required for melanoma vascularization

The above observations suggested a key role for Epac1 in the endothelium during melanoma growth and vascularization. However, global *Epac1* deletion does not distinguish endothelial effects from potential contributions of Epac1 expressed in other stromal cells which also contribute to angiogenesis and vascular remodelling.

**Fig. 2:**
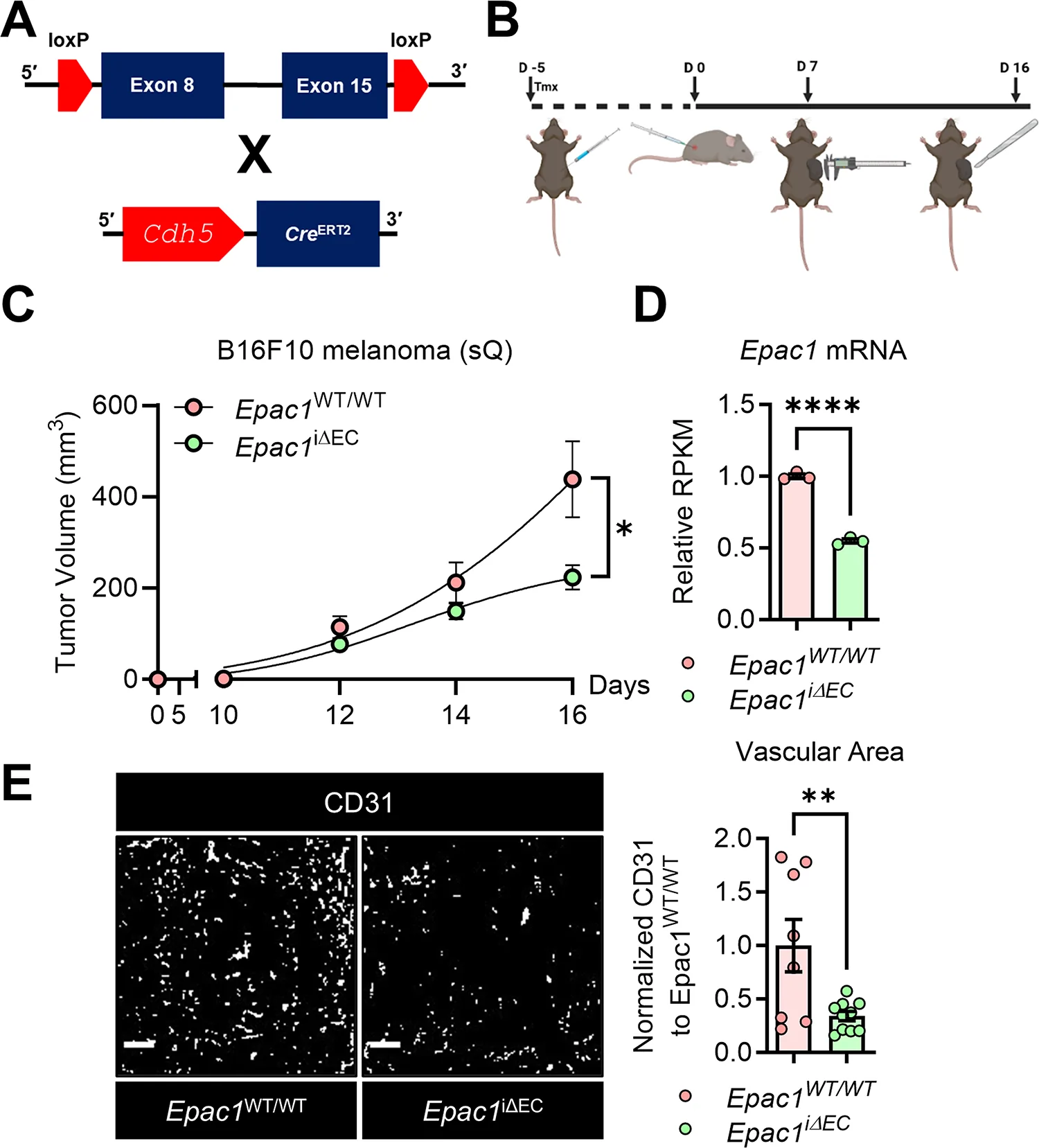
Endothelial-selective deletion of Epac1 decreases tumor growth and angiogenesis ** A**. *Epac1*^iΔEC^ mice were generated by crossbreeding *Epac1*^flox/flox^ with *Cdh5*-*Cre*^ERT2^ mice. A loxP site was inserted upstream of exon 8, and the frt-Neo-frt-loxp cassette was cloned downstream of exon 15. **B**. The *Epac1* endothelial-specific knockout was induced by treating mice with 75 mg/kg BW Tamoxifen (Tmx) in 100 µl corn oil intraperitoneally for five consecutive days. Following tamoxifen induction, mice received a single subcutaneous injection of 0.5 × 10⁶ B16F10 melanoma cells suspended in 100 µl PBS on the lateral flank. **C**. B16F10 melanoma tumor volume in *Epac1*^WT/WT^ and *Epac1*^iΔEC^ mice. The x-axis represents the days since tumor cell inoculation. Tumor growth was monitored using calliper measurements on days 10, 12, 14, and 16 post-inoculation; N = 20–22 mice per group. **D**. Quantification of *Epac1* mRNA level in TEC isolated from B16F10 tumors of *Epac1*^WT/WT^ and *Epac1*^iΔEC^ mice determined by normalized reads per kilobase per million mapped reads (RPKM). **E**. Representative images of tumor vascularization of B16F10 subcutaneous melanoma tumors excised from *Epac1*^WT/WT^ and *Epac1*^iΔEC^ mice. Vascularization was visualized with CD31 staining. The total vascular area was measured. Data represent the average CD31-positive area for each tumor and are the mean ± SEM of 8–10 mice per group and are expressed normalized to the average vascular area from *Epac1*^WT/WT^. Scale bars, 20 µm. * * p* < 0.05, **  * p*< 0.01, **** * p*< 0.0001.

To directly assess the endothelial contribution, we generated an inducible endothelial-specific *Epac1* KO (*Epac1*^iΔEC^) line by crossing *Epac1*^flox/flox^ with *Cdh5*-*Cre*^ERT2^ mice (Fig. 2A). Following subcutaneous B16F10 inoculation, tumor growth was monitored for 16 days (Fig. 2B). Consistent with our hypothesis, melanoma growth was significantly reduced in *Epac1*^iΔEC^ compared with *Epac1*^WT/WT^ mice (Fig. 2C). Endothelial *Epac1* deletion was confirmed in isolated tumor endothelial cells (TEC) and achieved an approximate knockout efficiency of 50% (Fig. 2D). Consistent with the reduced tumor volume, CD31 staining revealed a significantly smaller vascular area in *Epac1*^iΔEC^ tumors (Fig. 2E). These results demonstrate that endothelial Epac1 directly contributes to melanoma vascularization and growth.

## Endothelial *Epac1* deletion suppresses angiogenesis-related genes

**Fig. 3:**
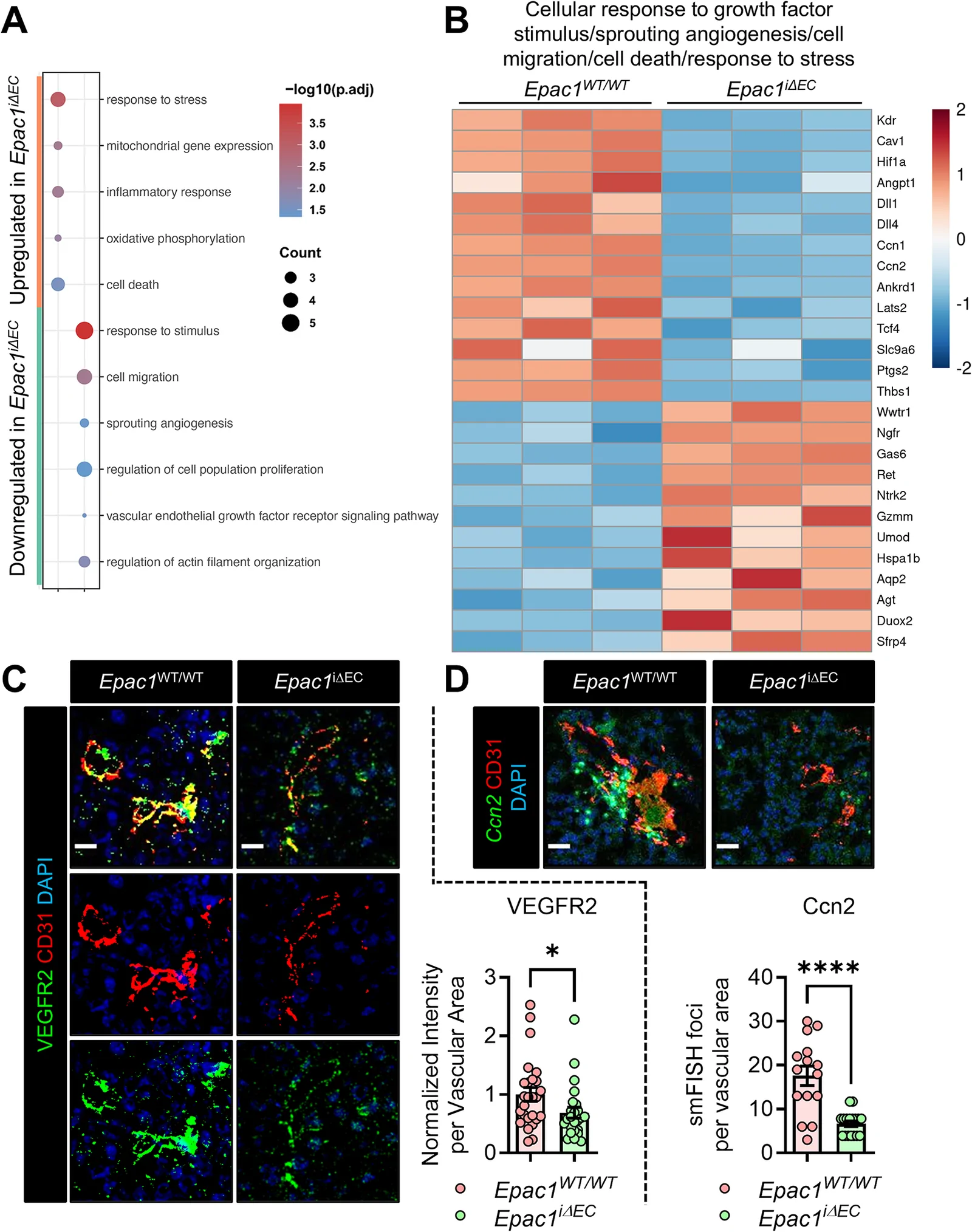
Endothelial-selective deletion of Epac1 in TEC attenuates YAP/TAZ and angiogenesis-related pathways ** A**. GO enrichment analysis of differentially expressed genes on upregulated (orange) and downregulated (green) genes in *Epac1*^iΔEC^ mice compared to *Epac1*^WT/WT^ mice in TEC of B16F10 subcutaneous melanoma tumors. Adjusted * p*-value < 0.05 and log₂fold change > 0 (upregulated) or < 0 (downregulated). **B**. Heatmap visualization of upregulated (red) and downregulated (blue) genes comprising cell migration, cellular response to growth factor stimulus and sprouting angiogenesis in TEC isolated from B16F10 subcutaneous melanoma tumor in *Epac1*^WT/WT^ and *Epac1*^iΔEC^ mice. Adjusted * p*-value < 0.05 and log₂fold change > 0 (upregulated) or < 0 (downregulated). **C**. Representative images of VEGFR2 (green) and CD31 (red) staining in sections of B16F10 subcutaneous melanoma tumors in *Epac1*^WT/WT^ and *Epac1*^iΔEC^ mice. Nuclei were counterstained with DAPI (blue). Normalized VEGFR2 per vascular area was quantified. Data represent VEGFR2 expressed as mean ± SEM from at least 3 animals. Scale bars, 20 µm. **D**. Representative images of *Ccn2* smFISH (green) and CD31 (red) staining in sections of B16F10 subcutaneous melanoma tumors in *Epac1*^WT/WT^ and *Epac1*^iΔEC^ mice. Nuclei were counterstained with DAPI (blue). *Ccn2* per vascular area was quantified. Data were normalized to the average vascular area and are presented as mean ± SEM of at least 3 animals. Scale bars, 20 µm. *  *p* < 0.05, **** *p* < 0.0001

To elucidate the molecular mechanisms underlying impaired melanoma angiogenesis in *Epac1*^iΔEC^ mice, TEC were isolated on day 16 post-tumor cell inoculation and subjected to whole transcriptome sequencing. Gene Ontology (GO) enrichment analysis revealed significant downregulation of angiogenesis-associated pathways, including sprouting angiogenesis, EC migration, and proliferation (Fig. 3A). Correspondingly, key pro-angiogenic genes such as *Kdr* (*Vegfr2*), *Angpt1*, *Hif1a*, *Dll1* and *Dll4* were reduced (Fig. 3B).

Notably, these enrichments included genes regulated by YAP/TAZ-dependent TEAD-family transcription factors—*Ccn1* (*Cyr61*), *Ccn2* (*Ctgf*) and *Ankrd1 (*Fig. 3B), suggesting dysregulation of YAP/TAZ activity. Notably, *Yap1* expression was unchanged, whereas *Wwtr1* transcripts were increased in TEC from Epac1-deficient mice. Because YAP/TAZ activity is primarily regulated through post-translational mechanisms and nuclear localization, transcript abundance was not used as a surrogate measure of YAP/TAZ activity. Consistent with the transcriptome data, VEGFR2 staining showed markedly decreased expression within the vascular area (Fig. 3C). Furthermore, single-molecule FISH for *Ccn2* demonstrated reduced transcript abundance in CD31-positive vascular regions of Epac1-deficient tumors (Fig. 3D). In human melanoma single-cell RNA-seq data, *CCN2* was detected in both endothelial cells and pericytes and was increased in tumor tissue compared to healthy skin (Fig. S3). These data indicate that *CCN2* is expressed across multiple vascular-associated cell types within the tumor microenvironment and that the observed reduction in tumor vascular regions following endothelial-specific Epac1 deletion likely reflects changes within the vascular compartment as a whole.

## Endothelial Epac1 regulates transcriptional activity of YAP/TAZ in vitro

**Fig. 4:**
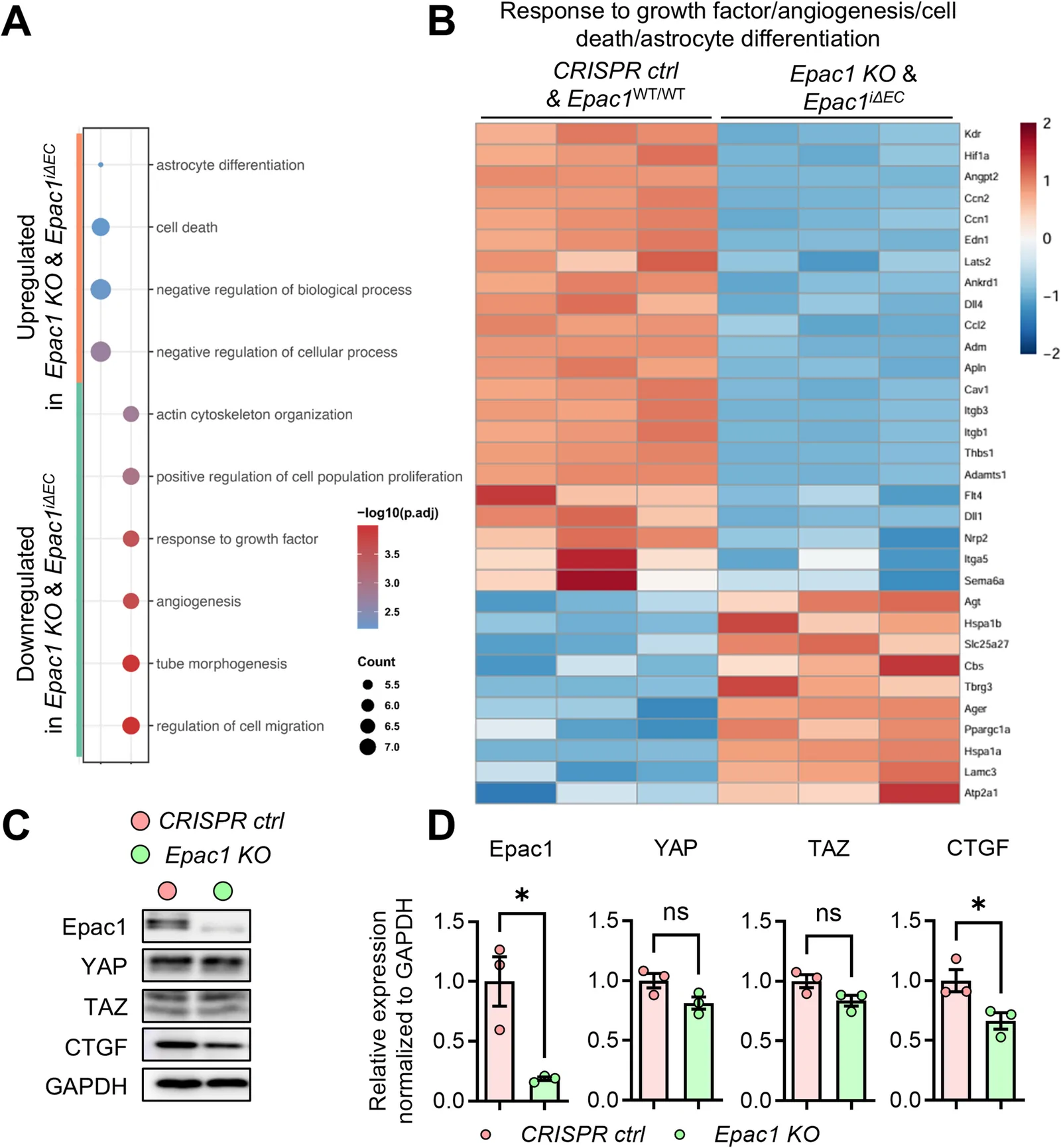
Epac1 deletion in EC disrupts YAP/TAZ and angiogenesis-related pathways ** A**. GO enrichment analysis of differentially expressed genes on upregulated (orange) and downregulated (green) genes shared between TEC isolated from B16F10 subcutaneous melanoma tumors from *Epac1*^WT/WT^ and *Epac1*^iΔEC^ mice and in CRISPR ctrl and *Epac1* KO bEnd.3 cells. Adjusted * p*-value < 0.05 and log₂fold change > 0 (upregulated) or < 0 (downregulated) for TEC, and > 0.58 (upregulated) or <  − 0.58 (downregulated) for bEnd.3. **B**. Heatmap visualization of upregulated (red) and downregulated (blue) genes comprising angiogenesis and response to growth factor between TEC isolated from B16F10 subcutaneous melanoma tumors from *Epac1*^WT/WT^ and *Epac1*^iΔEC^ mice and in CRISPR ctrl and *Epac1* KO bEnd.3 cells. Representative genes in upregulated (red) and downregulated (blue) genes are presented. Adjusted * p*-value < 0.05 and log₂fold change > 0 (upregulated) or < 0 (downregulated) for TEC, and > 0.58 (upregulated) or <  − 0.58 (downregulated) for bEnd.3. **C**. Representative western blot images of CRISPR ctrl and *Epac1* KO bEnd.3 cells and quantification of Epac1 protein expression. **D**. Quantification of YAP, TAZ and CTGF protein expression. Data are normalized to CRISPR ctrl and represent mean ± SEM of 3 independent experiments. *  * p* <0.05, n.s. * p*> 0.05.

To investigate the mechanism in vitro, CRISPR-cas9-mediated *Epac1* deletion was introduced in endothelial bEnd.3 cells (Fig. S4A-B). After confirming deletion efficiency (Fig. S4C-D), transcriptome sequencing of confluent cultures revealed that genes downregulated in both TEC from *Epac1*^iΔEC^ and *Epac1* KO bEnd.3 cells were enriched in GO terms related to angiogenesis, proliferation and migration (Fig. 4A). Shared downregulated genes included *Kdr*, *Hif1a*, and *Angpt2*, as well as canonical YAP/TAZ targets *Ccn2*, *Ccn1* and *Ankrd1* (Fig. 4B) thereby confirming that the impairment of YAP/TAZ activity found in *Epac1*^iΔEC^ TEC was maintained in vitro. YAP and TAZ protein levels were unchanged in *Epac1* KO cells (Fig. 4C-D), whereas CCN2 protein was decreased, indicating that Epac1 modulates YAP/TAZ transcriptional activity independent of their protein abundance. While Epac1 is best known as a GEF for Rap1, several studies indicated that it may also exert non-catalytic functions, such as acting as a scaffold for signalling complexes [50, 51]. To confirm these findings in a primary EC model, we employed human umbilical vein EC (HUVEC) that were treated with CE3F4, a selective and non-cyclic nucleotide-based inhibitor of Epac1 GEF activity [52]. CE3F4 similarly reduced expression of YAP/TAZ target genes (*CCN1*, *CCN2*, and *ANKRD1*) (Fig. S5A) in HUVEC, demonstrating that the enzymatic activity of Epac1 is required for maintaining YAP/TAZ-dependent transcription.

## Epac1 regulates YAP/TAZ nuclear localization

**Fig. 5:**
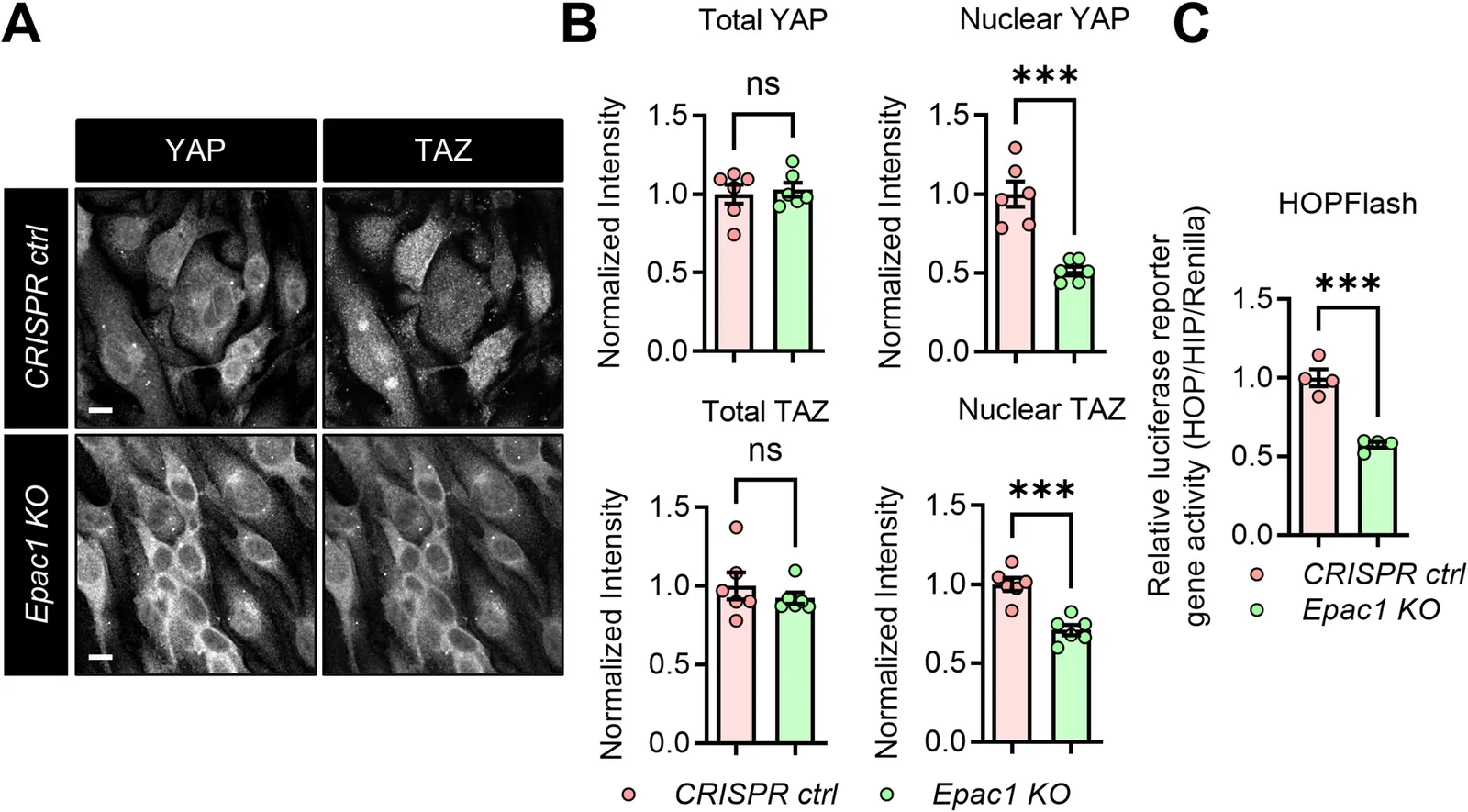
Endothelial Epac1 modulates YAP/TAZ nuclear localization and transcriptional activity ** A**. Representative images of YAP and TAZ staining in CRISPR ctrl and *Epac1* KO cells. Nuclei were counterstained with DAPI (not shown). Scale bars, 10 µm. **B**. Quantification of total and nuclear YAP and TAZ. Data are normalized to CRISPR ctrl and represent mean ± SEM of 8 independent experiments. **C**. TEAD luciferase reporter gene assay (HOPFlash) in CRISPR ctrl and *Epac1* KO cells. Cells were co-transfected with renilla luciferase and normalized to renilla luciferase expression. Data are normalized to non-specific reporter gene activity using a TEAD-mutant binding domain (HIPFlash) and compared to CRISPR ctrl and represent mean ± SEM of 4 independent experiments. *** * p*<0.001, n.s.* p* > 0.05

YAP and TAZ are central regulators of angiogenesis [53], whose activity is controlled by their subcellular localization. Immunostaining of CRISPR ctrl and *Epac1* KO cells revealed a significant reduction in nuclear YAP and TAZ in *Epac1*-deficient cells, while total protein levels remained unchanged (Fig. 5A-B). In line with these findings, TEAD reporter assays (7xTEAD minimal promoter sequence (HOPFlash) or its mutated version (HIPFlash) [43]), showed markedly reduced TEAD-dependent transcriptional activity in *Epac1* KO cells (Fig. 5C). Similarly, pharmacological inhibition of Epac1 with CE3F4 reduced YAP/TAZ nuclear translocation (Fig. S5B) in HUVEC.

## Epac1 coordinates VEGF/VEGFR2-mediated activation of YAP/TAZ signalling through RhoA-dependent regulation of the actin cytoskeleton

**Fig. 6:**
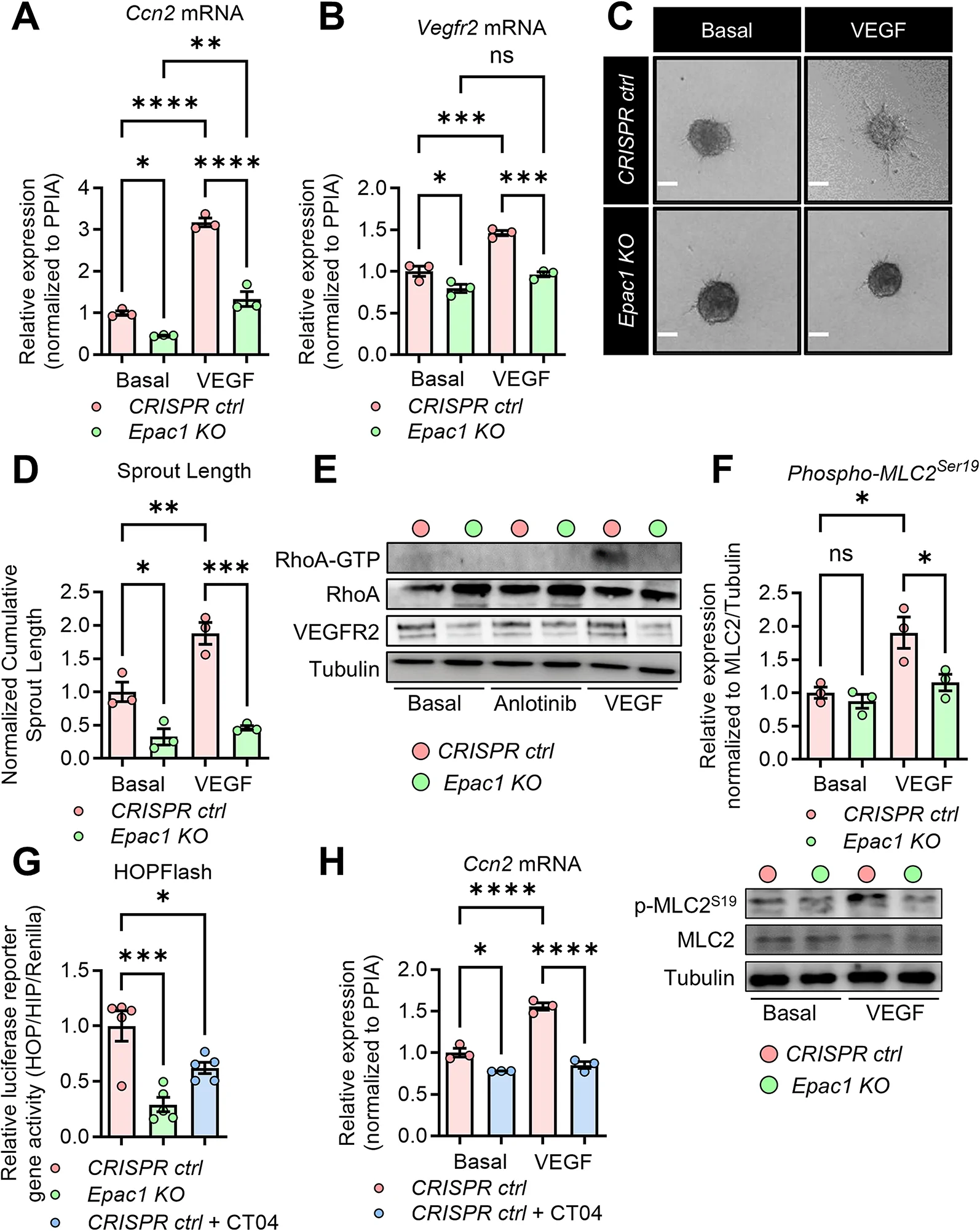
Endothelial Epac1 coordinates VEGF/VEGFR2 signalling through actin cytoskeleton** A**. *Ccn2* mRNA expression in CRISPR ctrl and *Epac1* KO bEnd.3 cells after VEGF (50 ng/ml, 30 min). Data are normalized to untreated CRISPR ctrl and represent mean ± SEM of 3 independent experiments. **B**. *Vegfr2* mRNA expression in CRISPR ctrl and *Epac1* KO bEnd.3 cells after VEGF (50 ng/ml, 30 min). Data are normalized to untreated CRISPR ctrl and represent mean ± SEM of 3 independent experiments. **C**. Representative images of endothelial sprouting assay in bEnd.3 CRISPR ctrl and *Epac1* KO cells. Spheroids were embedded in a collagen gel and incubated with VEGF (50 ng/ml, 24 h). **D**. Quantification of cumulative sprout length in CRISPR ctrl and *Epac1* KO cells incubated with VEGF (50 ng/ml, 24 h). Data represent the mean cumulative sprout length ± SEM of 3 independent experiments, each totalling 10–15 spheroids/condition. Scale bars, 100 µm. **E**. Representative western blot image of GTP-bound RhoA, total RhoA and VEGFR2 in CRISPR ctrl and *Epac1* KO bEnd.3 cells after VEGF (50 ng/ml, 30 min) or anlotinib (1 nM, 2 h) incubation. **F**. Representative western blot images and quantification of MLC2 expression and Ser19 phosphorylation of MLC2 in CRISPR ctrl and *Epac1* KO bEnd.3 cells after VEGF treatment (100 ng/ml, 10 min). Data are normalized to untreated CRISPR ctrl cells and represent mean ± SEM of 3 independent experiments. **G**. TEAD luciferase reporter gene assay (HOPFlash) in CRISPR ctrl and *Epac1* KO bEnd.3 cells. CRISPR ctrl was either untreated or treated with CT04 (2 µg/ml, 120 min). Cells were co-transfected with renilla luciferase and normalized to renilla luciferase expression. Data are normalized to non-specific reporter gene activity using a TEAD-mutant binding domain (HIPFlash) and compared to CRISPR ctrl and represent mean ± SEM of 4 independent experiments. **H**. *Ccn2* mRNA expression in bEnd.3 cells pre-incubation with CT04 (2 µg/ml, 2 h) and incubation with VEGF (50 ng/ml, 30 min). Data are normalized to untreated cells and represent mean ± SEM of 3 independent experiments. * p<0.05, ** * p*<0.01, *** * p*<0.001, **** * p*<0.0001, n.s. * p*>0.05.

Epac1 has previously been implicated in VEGFR2 expression [22], and VEGFR2-signalling is known to regulate YAP/TAZ activity in the vasculature. We therefore analysed YAP/TAZ-dependent gene expression in response to VEGF stimulation in *Epac1*-deficient cells. VEGF robustly induced *Vegfr2* and *Ccn2* expression in control cells, whereas this response was markedly reduced in Epac1-deficient cells, which also showed lower basal mRNA levels of both genes (Fig. 6A-B). Consistent with the reduced tumor vascularization observed in vivo, Epac1 deletion completely abolished both basal and VEGF-induced endothelial sprouting in vitro (Fig. 6C-D), including angiogenesis related cell proliferation (Fig. S5C). Similar results were obtained upon Epac1-inhibition in HUVEC where proliferation [54] and cell permeability (Fig. S5D) [55, 56] were affected. This impaired angiogenic response was accompanied by reduced endothelial cell proliferation in bEnd.3 after Epac1 deletion (Fig. S6). To confirm the dependence of YAP/TAZ activation on VEGF signalling, HOPFlash reporter gene and sprouting assays were performed in the presence of the VEGFR2 inhibitor anlotinib at a concentration that preferentially inhibits VEGFR2 [57]. Anlotinib blunted both endogenous and exogenous VEGF-induced sprouting (Fig. S7A-B) and strongly reduced TEAD-mediated transcription in control cells, while no additional effect was observed in *Epac1* KO (Fig. S7C). Mechanistically, VEGF signalling links to YAP/TAZ activity via changes in the cytoskeleton by activating RhoA/ROCK [53, 58]. Indeed, RhoA activation was increased by VEGF-signalling in control but not in *Epac1* KO cells (Fig. 6E). Consistently, phosphorylation of myosin light chain 2 (MLC2) at serine 19 (Ser19), a key downstream target of the RhoA effector kinase, ROCK [59, 60], was markedly reduced in *Epac1*-deficient cells (Fig. 6F). Pharmacological inhibition of RhoA with C3 transferase (CT04) significantly decreased TEAD-driven luciferase activity (Fig. 6G) and suppressed VEGF-induced *Ccn2* expression (Fig. 6H). Together these data indicate that Epac1 facilitates VEGF/VEGFR2-driven YAP/TAZ activation through RhoA-dependent cytoskeletal regulation.

## VEGF induced YAP/TAZ nuclear localization through RhoA/ROCK axis

**Fig. 7:**
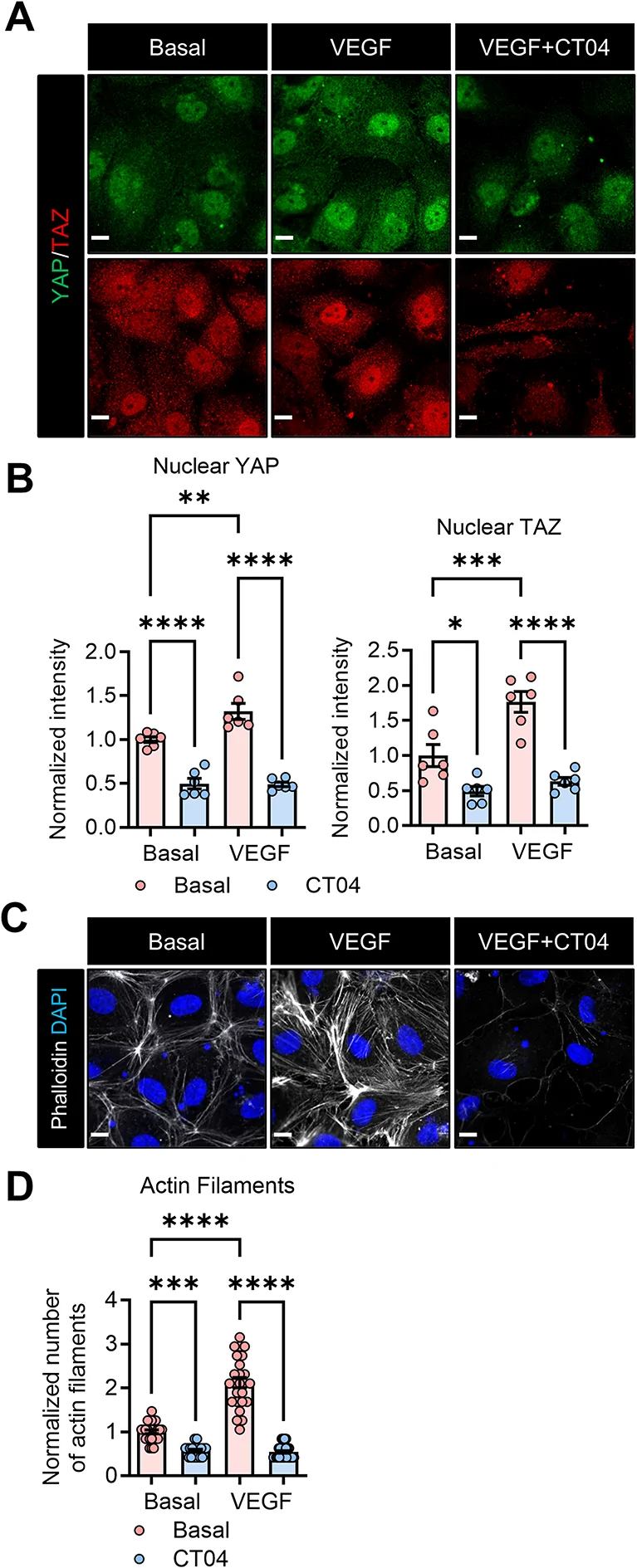
VEGF leads to YAP/TAZ nuclear localization dependent on Rho/ROCK-mediated cytoskeletal dynamics** A**. Representative images of YAP (green) and TAZ (red) staining in HUVEC after pre-incubation with CT04 (2 µg/ml, 2 h) and incubation with VEGF (50 ng/ml, 1 h). Nuclei were counterstained with DAPI (not shown). Scale bars, 10 µm. **B**. Quantification of nuclear YAP and nuclear TAZ in HUVEC. Data are normalized to Basal and represent mean ± SEM of 4 independent experiments. **C**. Representative images of phalloidin staining of actin filaments (gray) in HUVEC after pre-incubation with CT04 (2 µg/ml, 2 h) and incubation with VEGF (50 ng/ml, 1 h). Nuclei were counterstained with DAPI (blue). **D**. Actin filaments were quantified. Data are normalized to untreated cells and represent mean ± SEM of 4 independent experiments. Scale bars, 10 µm. * p<0.05, ** p<0.01, *** * p*<0.001, **** * p*<0.0001, n.s. * p*>0.05.

To assess whether VEGF signaling promotes YAP/TAZ transcriptional activity through the RhoA/ROCK pathway, HUVEC were stimulated with VEGF in the absence and presence of CT04. HUVEC were chosen as a human primary endothelial cell model to validate key mechanistic findings observed in murine EC. VEGF induced a marked increase in nuclear YAP (green) and TAZ (red) localization, which was fully prevented by RhoA inhibition (Fig. 7A-B). Likewise, VEGF-driven cytoskeletal reorganization was abolished by CT04 treatment (Fig. 7D). To confirm that these findings are not restricted to human primary EC, similar experiments were repeated in bEnd.3 cells, which showed consistent results (Fig. S8A-B). Together, these data confirm that VEGF-mediated activation of YAP/TAZ depends on RhoA/ROCK-driven cytoskeletal dynamics, likely through stress fiber-dependent actomyosin tension.

## Loss of *Epac1* impairs mechanosensing and mechanoregulation of endothelium

Tumor vessels are typically irregular and poorly organized [1–4, 61], leading to disturbed blood flow, hypoxia, acidosis, and elevated interstitial fluid pressure [5, 62]. Disturbed flow alters endothelial mechanosensing and promotes pro-angiogenic signalling, including YAP/TAZ signalling, and metastasis [49, 63]. Given that Epac1 regulates VE-cadherin dynamics, [55, 56], we hypothesized that it may facilitate VEGFR2/VE-cadherin interactions within the endothelial mechanosensory complex.

**Fig. 8:**
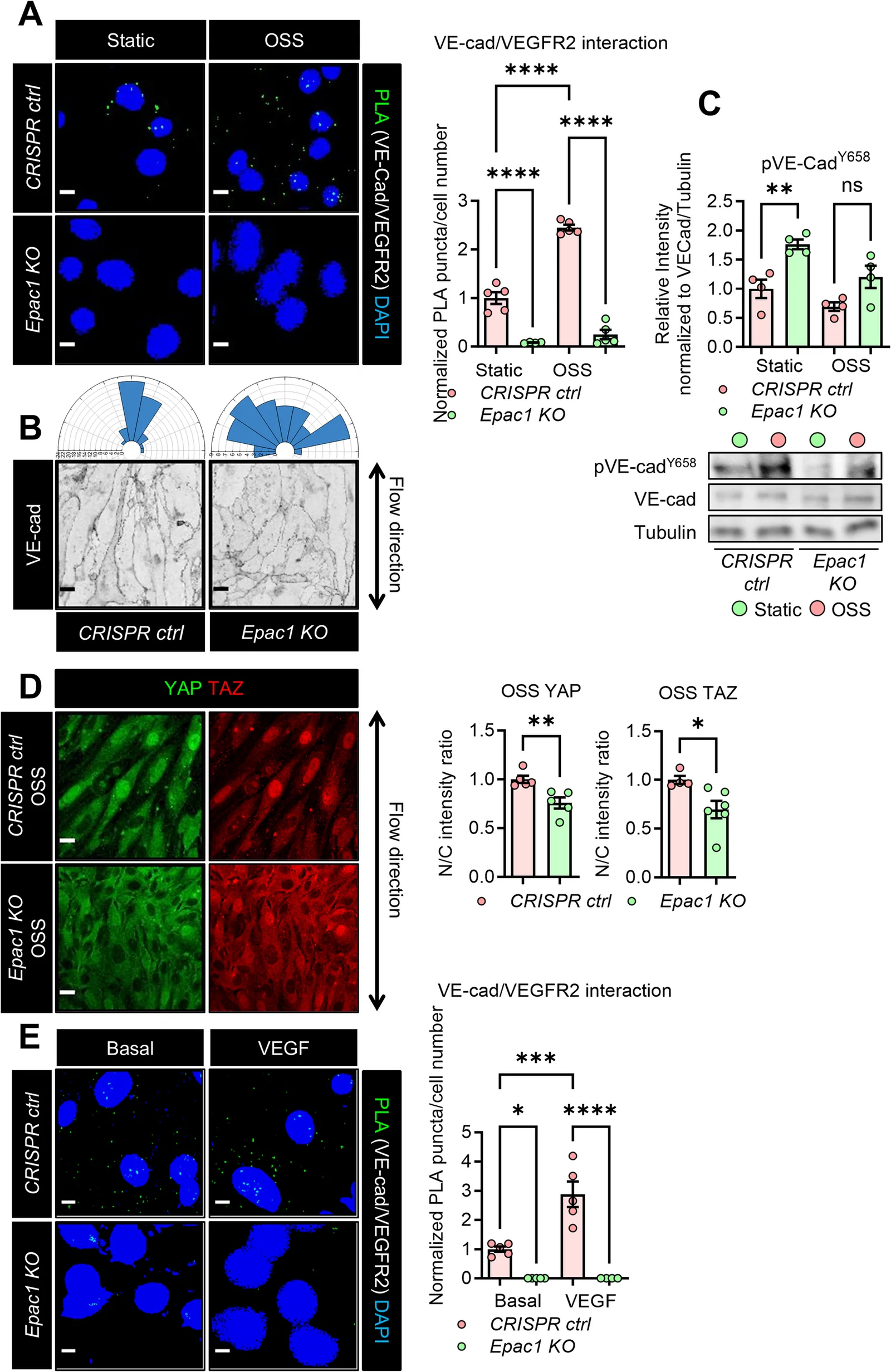
Interaction of VEGFR2 and VE-cadherin is required for shear stress-induced YAP/TAZ **A**. Representative proximity ligation assay images of VEGFR2 and VE-cadherin in CRISPR ctrl and *Epac1* KO cells under oscillatory shear stress (OSS, 12 dyne/cm^2^ at 2 Hz) and static conditions. Nuclei were counterstained with DAPI (blue). Data are normalized to static CRISPR ctrl cells and represent mean ± SEM of 3 independent experiments. Scale bars, 10 µm. **B**. Representative images of VE-cadherin staining in CRISPR ctrl and *Epac1* KO cells under oscillatory shear stress (OSS, 12 dyne/cm^2^ at 2 Hz). Windrose plot showing the direction of EC alignment. Scale bars, 10 µm. **C**. Representative western blot images and quantification of VE-cadherin expression and Y658 phosphorylation in CRISPR ctrl and *Epac1* KO cells under oscillatory shear stress (OSS, 12 dyne/cm^2^ at 2 Hz) and static conditions. Data are normalized to static CRISPR ctrl cells and represent mean ± SEM of 4 independent experiments. **D**. Representative images and quantification of YAP (green) and TAZ (red) staining in CRISPR ctrl and *Epac1* KO cells under oscillatory shear stress (OSS, 12 dyne/cm^2^ at 2 Hz). Nuclei were counterstained with DAPI (not shown) to determine the nuclear area. Nuclear-to-cytosolic ratios (N/C ratio) of YAP and TAZ in static and OSS conditions were quantified and normalized to CRISPR ctrl cells. Scale bars, 10 µm. **E**. Representative proximity ligation assay images of VEGFR2 and VE-cadherin (green) in CRISPR ctrl and *Epac1* KO cells after VEGF (50 ng/ml, 6 h). Nuclei were counterstained with DAPI (blue). Data are normalized to untreated CRISPR ctrl cells and represent mean ± SEM of 3 independent experiments. * p < 0.05, ** *p*< 0.01, **** p < 0.0001, n.s.* p* > 0.05. Scale bars, 10 µm.

Using oscillatory shear stress (OSS) to mimic disturbed flow [62, 64–66], we found that OSS markedly increased VE-cadherin/VEGFR2 interaction, as detected by proximity ligation, whereas this interaction was abolished by *Epac1*-deficiency (Fig. 8A). Under OSS, control cells aligned in the direction of the flow, whereas *Epac1* KO cells failed to do so (Fig. 8B).

OSS also induced phosphorylation of VE-cadherin at Y658—an essential modification for mechanosensing [67, 68]—which was markedly reduced in *Epac1* KO cells (Fig. 8C). Pharmacological inhibition of VEGFR2 with anlotinib similarly disrupted flow-induced alignment (Fig. S9). Correspondingly, *Epac1* loss prevented OSS-induced nuclear localization of YAP and TAZ (Fig. 8D). Accordingly, VEGF stimulation induced VEGFR2/VE-cadherin interaction in control, but not *Epac1*-deficient cells (Fig. 8E). Finally, lentiviral re-expression of Epac1 in *Epac1* KO cells (Fig. S4C-E), restored *Vegfr2* and *Ccn2* expression (Fig. S10A), and rescued responses to VEGF (Fig. S10B-C) and OSS (Fig. S10D-E). The results confirm that Epac1 is essential for VEGFR2/VE-cadherin complex formation and for endothelial mechanosensing.

## Discussion

In the present study, we elucidate the role of endothelial Epac1 in the regulation of tumor growth and angiogenesis. Since its discovery in 1998, Epac1 has been recognized as a key regulator of both physiological and pathological vascular adaptation [22, 55, 56, 69]. In this context, Epac1 has been established as an important modulator of pro-angiogenic pathways, including VEGF/VEGFR2 signaling, while global pharmacological Epac inhibition was shown to impair tumor growth and angiogenesis [16]. In contrast, our study addresses the specific function of the endothelial-expressed isoform Epac1 during melanoma vascularization, identifying Epac1 as the major driver of VEGF/VEGFR2-YAP/TAZ-mediated tumor progression.

In EC, Epac1 is essential for cell migration [22, 69], proliferation [69], and junctional remodelling [55, 56], all of which are key mechanisms underlying angiogenesis. As a downstream effector of GPCRs, Epac1 can be activated by pathological stimuli such as prostaglandins [70, 71], and catecholamines [72], both abundantly secreted in the tumor microenvironment. In contrast, previous work by Liu et al. [69] and our group [22] independently demonstrated that Epac1 is dispensable in physiological angiogenesis. At postnatal day 5, the retinal vascular area was comparable between *Epac1*-deficient and wild-type mice. Interestingly, when subjected to oxygen-induced retinopathy (OIR), pre-retinal neovascularization was significantly reduced in *Epac1*^KO/KO^ animals, indicating a critical role for Epac1 in pathological—but not physiological—angiogenesis [22, 69]. This finding underscores the mechanistic divergence between pathological and physiological vascular growth, where negative-feedback regulators such as Notch signalling limit excessive vessel sprouting [73, 74].

Collectively, our findings highlight that Epac1 functions as a context-dependent regulator of angiogenesis rather than as a uniformly pro- or anti-angiogenic factor. The divergent outcomes reported in the literature [21] may reflect fundamental differences between physiological and pathological angiogenesis. Whereas sustained pharmacological activation of Epac1 can induce anti-angiogenic signaling, our data indicate that endogenous endothelial Epac1 is required for efficient VEGF-driven signaling and pathological angiogenesis in melanoma and OIR [22]. Thus, Epac1 may be largely dispensable during physiological angiogenesis and vascular homeostasis while becoming functionally indispensable in pathological settings characterized by excessive VEGF signaling and altered mechanical cues.

Here, we propose a new role for Epac1 as a driver of pathological melanoma angiogenesis. The scRNA-seq analysis confirmed that Epac1 is highly expressed in human melanoma EC compared to other cell types, and its expression is significantly elevated relative to endothelium from healthy skin. Previously, we demonstrated that Epac1 regulates endothelial VEGFR2 expression in vitro [22]. Consistent with this finding, endothelial *Epac1*-deficiency in vivo resulted in attenuated melanoma angiogenesis, characterized by decreased VEGFR2 levels, critical for sensing VEGF gradients and sustaining VEGF responsiveness through a positive-feedback loop [5, 74–76]. Deletion of *Vegfr2* in mice similarly results in tumor vessel regression and impaired tumor growth [77], consistent with the established role of VEGFR2 as a central factor of tumor-associated vascular growth and a hallmark of malignant progression.

VEGFR2 signalling depends on proper receptor localization at the cell membrane [78, 79]. Beyond transcriptional regulation, VEGFR2 levels are dynamically controlled through ligand-induced internalization, recycling, and degradation [80]. These events are influenced not only by VEGF [81] and VE-cadherin [82, 83] but also by cytosolic transport proteins involved in trafficking from the *trans*-Golgi network, including MACF1 [84] and MYO1C [85]. Interestingly, both MACF1 and MYO1C were downregulated upon *Epac1* deletion in bEnd.3 cells, suggesting that Epac1 may regulate VEGFR2 membrane trafficking. Similarly, downregulation of YAP/TAZ signalling, either by genetic knockout [53] or pharmacological inhibition [86], has been shown to impair VEGFR2 membrane localization, indicating a feedback loop between VEGFR2 and YAP/TAZ signalling. Such a mechanism may contribute to the complete loss of VEGFR2/VE-cadherin interaction observed at the plasma membrane of *Epac1* KO cells, despite only partial reduction of VEGFR2 expression. Nevertheless, the rapid downregulation of YAP/TAZ-dependent genes following pharmacological inhibition of Epac1 suggests that additional, more direct mechanisms may underlie Epac1-mediated facilitation of VEGF responsiveness. These mechanisms likely extend beyond transcriptional regulation and receptor localization to include modulation of vesicular transport proteins and cytoskeletal dynamics that maintain VEGFR2 availability at the cell membrane.

While our findings do not imply a direct physical interaction between Epac1 and YAP/TAZ, they suggest a more integrated role for Epac1 in sustaining endothelial sensitivity to angiogenic cues—most likely through its combined effects on VEGFR2 expression, trafficking, and the downstream YAP/TAZ signalling axis [53].

## Limitations

This study employed melanoma as a tumor model and although this is a highly vascularized tumor, results might not reflect tumor angiogenesis in other tumor models, both with regard to tumor growth and the prominent upregulation of Epac1 in tumor-associated vascular endothelium.

## Conclusions

In summary, the results from our study identify endothelial Epac1 as a crucial regulator of tumor growth and angiogenesis. We demonstrate that endothelial Epac1 governs VEGFR2 expression and signalling, and that its loss disrupts responsiveness to VEGF and mechanical stimuli, resulting in impaired YAP/TAZ-dependent transcriptional regulation. Collectively, these findings establish Epac1 as an essential component of the endothelial machinery that coordinates VEGF signalling and mechanotransduction during pathological tumor angiogenesis.

## Supplementary Information

Below is the link to the electronic supplementary material.

Supplementary Material 1

## Data Availability

Raw RNA-seq from TEC generated in this study were deposited in Gene Expression Omnibus (GEO) database under accession number GSE341674. Processed RNA-seq data were provided in the Supplementary Information.
