# Supplementary material for: Endothelial Epac1 facilitates YAP/TAZ controlled melanoma growth and angiogenesis
Source: Angiogenesis. 2026 Sep 23;29(4):72. doi: 10.1007/s10456-026-10080-6 (PMC13601084; doi:10.1007/s10456-026-10080-6)
Supplement: Supplementary file 1 [file 10456_2026_10080_MOESM1_ESM.docx]

Figure S1. **Expression of EC markers in scRNA-seq datasets.** A. UMAP of all cells in healthy human skin and primary human melanoma tissue analysed by scRNA-seq. Purple represents expression of vascular EC markers *CDH5* (left), *PECAM1* (middle), and *VWF* (right). B. UMAP of all cells in healthy human skin and primary human melanoma tissue analysed by scRNA-seq. Purple represents expression of lymphatic EC markers *LYVE1* (left), *PROX1* (middle), and *FLT4* (right). C. *RAPGEF3* expression in EC vs all non-EC cells in healthy skin and melanoma. **** p<0.0001.

Figure S2. **Cell type annotation by canonical marker gene expression in healthy skin and melanoma scRNA-seq datasets.** A. UMAP with full cell type annotation of healthy skin (left) and melanoma (right) scRNA-seq datasets based on typical marker gene expression. B. Dot plot showing expression of known cell-specific marker genes in the scRNA-seq healthy skin clusters (Fig. S2A). Dot color intensity represents average expression of the gene in a given cluster, and dot size the percentage of cells in the cluster expressing that particular marker. C. Dot plot showing expression of known cell-specific marker genes in the scRNA-seq melanoma clusters (Fig. S2A). Dot color intensity represents average expression of the gene in a given cluster, and dot size the percentage of cells in the cluster expressing that particular marker.

Figure S3. ***CCN2* expression in EC and pericytes of healthy skin and melanoma*.*** Violin plots of *CCN2* expression in EC (left) and pericytes (right) of healthy skin and melanoma. **** p<0.0001.

Figure S4. ***Epac1* deletion in bEnd3 cells**. A. Representative images of bEnd.3 cells after lentiviral transduction with *Epac1* sgRNAs against mouse *Epac1* cloned in TLCV2 plasmid. Cells were treated by Doxycycline (Dox, 2 µg/ml) for 48h to select transduced cells. Images are bEnd.3 with sgRNA *Epac1* L1 (top) and sgRNA *Epac1* L4 (bottom) in brightfield (BF, left), FITC channel (green, middle) and Merged (right). Scale bars, 20 µm. B. Validation of knockout using mutation detection kit. The PCR products isolated from sgRNA *Epac1* transduced bEnd.3 were subjected to Resolvase-mediated cleavage of heteroduplex PCR products. Representative agarose gel showing cleavage activity from sgRNA *Epac1* transduced bEnd.3 compared to CRISPR ctrl. C. Western blot images of Epac1 expression in bEnd.3 transduced with CRISPR ctrl and sgRNAs against *Epac1.* Western blot image of Epac1 expression in bEnd.3 transduced with *Epac1* cloned in ADR3 IRES plasmid. D. Quantification of *Epac1* mRNA expression in bEnd.3 cells transduced with CRISPR ctrl and sgRNA *Epac1*. E. Representative western blot images of Epac1 expression in CRISPR ctrl, *Epac1* KO and *Epac1* KO *Epac1* overexpression (OE) bEnd.3 cells. Data are normalized to CRISPR ctrl and represent mean ± SEM of 3 independent experiments. * p<0.05, ** p<0.01.

Figure S5. **CE3F4 decreases YAP/TAZ target gene expression.** A. *CCN1, CCN2* and *ANKRD1* mRNA expression in HUVECs after CE3F4 (20 µM, 24h). Data are normalized to untreated HUVECs and represent mean ± SEM of 4 independent experiments. B. Representative images and quantification of YAP/TAZ (green) staining in HUVECs after CE3F4 (20 µM, 24h) treatment. Nuclei were counterstained with DAPI (not shown). Scale bars, 10 µm. C. Quantification of Ki67 positive cells in HUVECs after pre-incubation with CE3F4 (20 µM, 14 h). Data are normalized to the basal condition and represent mean ± SEM of 3 independent measurements. D. Quantification of endothelial permeability assay. HUVECs were pre-incubated with CE3F4 (20 µM, 14 h) after which FITC-dextran was added to the top compartment. Fluorescence was measured in the bottom compartment. Data are normalized to the basal condition and represent mean ± SEM of 3 independent measurements. * p<0.05, ** p<0.01, *** p<0.001, n.s. p>0.05.

Figure S6. ***Epac1* deletion prevents bEnd.3 proliferation.** Representative images of Ki67 (green) staining in CRISPR ctrl and *Epac1* KO cells. Nuclei were counterstained with DAPI. Ki67 positive cells per area were quantified and normalized to CRISPR ctrl cells. Scale bars, 10 µm. ** p<0.01.

Figure S7. **VEGFR2 inhibition and *Epac1* deletion prevent VEGF-induced sprouting**. A. Representative images of endothelial sprouting assay in bEnd.3 cells. Spheroids were embedded in a collagen gel and pre-incubated with anlotinib (1 nM, 1h) and incubated with VEGF (50 ng/ml, 24h). B. Quantification of cumulative sprout length in bEnd.3 cells pre-incubated with anlotinib (1 nM, 1h) and incubated with VEGF (50 ng/ml, 24h). Data represent mean cumulative sprout length ± SEM of 3 independent experiments, each totalling 10-15 spheroids/condition. Scale bars, 100 µm. C. TEAD luciferase reporter gene assay (HOPFlash) in CRISPR ctrl and *Epac1* KO bEnd.3 cells after anlotinib (1 nM, 48h). Cells were co-transfected with renilla luciferase and normalized to renilla luciferase expression. Data are normalized to non-specific reporter gene activity using a TEAD-mutant binding domain (HIPFlash) and compared to CRISPR ctrl and represent mean ± SEM of 3 independent experiments. * p<0.05, **** p<0.0001, n.s. p>0.05.

Figure S8. **VEGF leads to YAP/TAZ nuclear localization dependent on Rho/ROCK-mediated cytoskeletal dynamics in bEnd.3 cells.** A. Representative images of YAP (green) and TAZ (red) staining and quantification of nuclear localization in bEnd.3 cells after pre-incubation with CT04 (2 µg/ml, 2h) and incubation with VEGF (50 ng/ml, 1h). Nuclei were counterstained with DAPI (not shown). Scale bars, 10 µm. Data are normalized to Basal and represent mean ± SEM of 4 independent experiments. B. Representative images and quantification of phalloidin staining of actin filaments (gray) in bEnd.3 cells after pre-incubation with CT04 (2 µg/ml, 2h) and incubation with VEGF (50 ng/ml, 1h). Nuclei were counterstained with DAPI (blue). Actin filaments were quantified. Data are normalized to untreated cells and represent mean ± SEM of 4 independent experiments. Scale bars, 10 µm. ** p<0.01, **** p<0.0001.

Figure S9. **VEGFR2 inhibition prevents endothelial cell alignment in response to shear stress**. Windrose plot showing bEnd.3 cell alignment under static conditions and in response to oscillatory shear stress (OSS, 12 dyne/cm^2^ at 2 Hz). Cells were pre-incubated with anlotinib (1 nM, 24 h) prior to shear stress exposure (24h exposure).

Fig S10. **The re-expression of human Epac1 in *Epac1* KO bEnd.3 cells rescues impaired VEGF/VEGFR2 signalling**. A. *Epac1, Vegfr2 and Ccn2* mRNA expression in CRISPR ctrl, *Epac1* KO and *Epac1* KO *Epac1* OE bEnd.3 cells. Data are normalized to CRISPR ctrl and represent mean ± SEM of 3 independent experiments. B. Quantification of proximity ligation assay between VEGFR2 and VE-cadherin from CRISPR ctrl, *Epac1* KO and *Epac1* KO *Epac1* OE bEnd.3 cells under basal conditions and after VEGF (50 ng/ml, 6h). Nuclei were counterstained with DAPI (blue). Data are normalized to CRISPR ctrl basal and represent mean ± SEM of 3 independent experiments. C. qPCR for *Ccn2* mRNA expression of CRISPR ctrl, *Epac1* KO and *Epac1* KO *Epac1* OE bEnd.3. Cells were seeded and pre-starved with 0.5% FBS in EBM for 48h. Data are normalized to CRISPR ctrl and represent mean ± SEM of 3 independent experiments. D. Quantification of proximity ligation assay between VEGFR2 and VE-cadherin from CRISPR ctrl, *Epac1* KO and *Epac1* KO *Epac1* OE bEnd.3 cells under static conditions and in response to oscillatory shear stress (OSS, 12 dyne/cm^2^ at 2 Hz). Data are normalized to CRISPR ctrl under static conditions and represent mean ± SEM of 3 independent experiments. E. Representative images of VE-cadherin staining in CRISPR ctrl, *Epac1* KO, and *Epac1* KO *Epac1* OE bEnd.3 cells under static conditions and in response to oscillatory shear stress (OSS, 12 dyne/cm^2^ at 2 Hz). Scale bars, 10 µm. * p<0.05, *** p<0.001, **** p<0.0001, n.s. p>0.05
